# Trends of adverse pregnancy outcomes and its determinants in Arba Minch Zuria and Gacho Baba Woredas from 2018 to 2022: Analysis of health and demographic surveillance data

**DOI:** 10.1371/journal.pone.0313564

**Published:** 2025-01-16

**Authors:** Zeleke Gebru, Fekadeselassie Berhe, Shitaye Shibiru, Bereket Honja, Mesfin Kote, Alazr Baharu, Tadesse Awoke

**Affiliations:** 1 School of Public Health, College of Medicine and Health Sciences, Arba Minch University, Arba Minch, Ethiopia; 2 Hawassa University Comprehensive Specialized Hospital, Hawassa University, Hawassa, Ethiopia; 3 School of Nursing, College of Medicine and Health Sciences, Arba Minch University, Arba Minch, Ethiopia; 4 Hawassa Health Science College, Hawassa, Ethiopia; 5 Arba Minch Health and Demographic Surveillance Site (HDSS), Arba Minch, Ethiopia; 6 Department of Epidemiology and Biostatistics, Institute of Public Health, College of Medicine and Health Sciences, University of Gondar, Gondar, Ethiopia; Madda Walabu University, ETHIOPIA

## Abstract

**Introduction:**

Many family members and the expectant mother view pregnancy as a time of joyful anticipation. However, it can also bring about a range of issues that may pose serious and potentially life-threatening risks for both the mother and the unborn child. Adverse pregnancy outcomes are an alarming public issue in different parts of the world and have a seriously harmful influence on both their health and well-being. Nevertheless, less is known about trends and determinates of adverse pregnancy outcomes in the Arba Minch zuria and Gacho Baba districts in southern Ethiopia. Therefore, this study aimed to fill these gaps in the study setting.

**Methods:**

An open, dynamic cohort study design was employed among 8885 pregnancies from 2018 to 2022, health and demographic surveillance data were involved. Data to be collected at Arba Minch Health and Demographic Surveillance Site (HDSS) using a structured questionnaire. A log-binomial regression model was used to identify determinates. A P-value of less than 0.05 was considered to declare a statistically significant association.

**Results:**

In this study, the overall adverse pregnancy outcomes were 8.89 per 1000 live births [95%CI: 6.93, 10.84]. The stillbirth and abortion rates were 5.74 per 1000 live births (95%CI: 4.36, 7.54) and 3.15 per 1000 live births [95%CI: 1.97, 4.02], respectively. The trends of adverse pregnancy outcomes showed 11.1/1000 in 2018 and 14.1/1000 in 2022. Age > 34 years old (aPPR = 2.93, 95%CI: 1.67, 5.17), antenatal care (aPPR = 0.52, 95%CI: 0.33, 0.83), and history of pregnancy loss (aPPR = 2.68, 95%CI: 1.36, 5.29) were identified as determinates for adverse pregnancy outcomes.

**Conclusion:**

The prevalence of adverse pregnancy outcomes is still high, and trends vary from time to time. As such, attention is needed for the women who had a previous history of pregnancy loss, and addressing those determinants could potentially reduce the rates of stillbirths and abortions, ultimately promoting healthier pregnancies and better pregnancy outcomes.

## Introduction

Pregnancy is a natural phenomenon that involves the development and growth of the fetus inside the mother`s uterus. Most family members and the woman herself consider gestation as an occasion of joyous excitement and anticipation. Still, it can also be accompanied by various problems that can have serious and life-threatening complications for the mother as well as the unborn child. Adverse pregnancy outcomes may have a serious harmful influence on both their health and well-being [1]. When referring to an adverse pregnancy outcome, it encompasses serious complications and other health issues that can distress the newborn, the mother, or both at the time of pregnancy, labor, and the postpartum period, including neonatal death, preterm birth, stillbirth, under [low] birth weight, abortion, congenital deformity, and the death of a mother [2]. Despite the improvements in medical technologies and enhancements in access to healthcare services, adverse pregnancy outcomes are a prevalent problem, especially in low-middle-income countries, compared to high-income countries in the world. For instance, low-income nations have greater rates of stillbirth, abortion, and miscarriage [3–5]. Adverse pregnancy outcome prevalence varies from place to place in the world, according to different literature. In Sub-Saharan Africa, the overall prevalence was 29.7% [6]; in Saudi Arabia miscarriage was 12.1% [7], in India, the overall prevalence was 12% [8], and in Ethiopia, it ranges from 17.88% to 31.8% [9–11]; and in America, fetal death was 2.97% [12]. This evidence indicates adverse pregnancy outcomes are very common throughout the world.

Adverse pregnancy outcomes are strongly interlinked with socioeconomic level. According to research that appeared in the International Journal for Equity in Health, having a low socioeconomic condition increases the probability of experiencing a bad pregnancy outcome [13]. Unwanted pregnancy results, including stillbirth, miscarriage, and abortion, can undesirably influence and affect a woman’s mental, social, physical, and emotional health, and the family`s health as well. These impacts can have elongated consequences on women`s future gestations and reproductive health. A pregnant woman who experiences such pregnancy-related problems is prone to depression, post-traumatic sickness, and anxiety, as indicated in the literature [11]. Furthermore, this can also directly show the health care quality and socioeconomic status [13–15].

Different determinants are related to adverse outcomes in pregnant mothers. Low socioeconomic standing, complications related to pregnancy, and chronic illness are usually the main determinants of adverse pregnancy outcome outcomes. In a research study carried out in Ethiopia [10, 11], Asia [8], and America [12], it was revealed that women who experienced pregnancy problems were prone to having a poor delivery outcome, and the determinants that affect delivery and its consequences were identified. It was found that the primary factor in newborn and maternal illness and death was adverse pregnancy outcomes. It is essential to find these determinants and take appropriate measures to prevent those adverse pregnancy outcomes. Increasing access to health institutions, developing strategies to prevent and manage complications with current knowledge, and delivering focused antenatal care follow-up for pregnant women are recommended strategies [13].

Despite the existence of health and demographic surveillance in the area, research has never been done regarding unfavorable pregnancy consequences and factors that contributed to this outcome result. Additionally, only a few longitudinal studies have been carried out in our country. Therefore, the purpose of this study was to evaluate the adverse pregnancy outcomes and risks associated with it in Arba Minch Health and Demographic.

## Methods and materials

### Study settings

The data was collected at Arba Minch Health and Demographic Surveillance Site (HDSS), from 01, January 01, 2018, to 31, December, 2022. The data was collected from 12, July 2023 to 13, September 2023. The Health and Demographic Surveillance System (HDSS) unit is responsible for monitoring the demographic and health characteristics of a population residing in Arba Minch Zuria woreda and Gacho Baba woreda in Gamo Zone. The HDSS began with an initial census, followed by regular updates on key demographic events (such as births, deaths, and migrations) and health events twice a year. Operating in nine Kebeles of Arba Minch Zuria woreda and Gacho Baba woreda in Gamo Zone, the program comprises two components: demographic surveillance and mortality surveillance. Since its inception in 2009, the program has conducted a census and continues to update events biannually. Kebeles are the smallest administrative unit (defined as the smallest administrative unit of Ethiopia, and it is a neighborhood or a localized and delimited group of people). During the biannual field visits, data collectors ensure the household inhabitant list is up to date. The questionnaire used during these visits covers information on house characteristics, births, deaths, migrations, pregnancies, pregnancy outcomes, and vaccination status.

The area had 14,754 houses and 74,107 residents (males 37,130 and females 36,977), according to the report on the area. The population rose by an average of 2.33% year between 2009 and 2014–15. The age range of 25 to 29 had the greatest fertility rate in 2015 [209.3/1,000], followed by the age range of 30–34 [203.6/1,000], according to the report. The lowest prevalence was recorded in the 45–49 age group [36.9/1000], while it was 64/1,000 births for teenagers [14–18]. The average female fertility rate over six years was 53.6%. The area is well known for its cash crops, including bananas, mangoes, avocados, and vegetables.

### Study design and period

An open, dynamic cohort study design that longitudinally monitors well-specified primary subjects and all associated socio-demographic and health-related outcomes within a precisely defined geographic area. The event records (data) of women whose pregnancies were registered between 2018 and 2022 were included.

### Population and selection criteria

The study population included all mothers who lived in Arba Minch HDSS and had pregnancies that ended between 2018 and 2022. Data with missing outcome variables were not included in the analysis.

### Sample size and sampling procedure

Data was extracted from the Arba Minch HDSS database system for a period of about five years, from 2018 to 2022. During the study period, every household with a mother who had ever been pregnant and experienced a pregnancy outcome was taken into account. All pregnancies, about 8885 pregnancies noted in the HDSS site database between 2018 and 2022, were included in the study. Based on the nine kebeles proximity to Arba Minch Town, climate zone, and degree of urbanization, they were picked as locations for the demographic surveys. For this study, all pregnancies b/n 2018 and 2022 having birth comes outcomes included.

### Operational definition

An adverse pregnancy outcome/ history of pregnancy loss in this study implies the existence of at least one or more events, including stillbirth, miscarriage, and fetal loss [16] in the pregnancies between 2018 and 2022. The World Health Organization (WHO) defined abortion as the intentional termination of a pregnancy, most frequently performed during the first 28 weeks of pregnancy. When a fetus passes away before 28 weeks of pregnancy without any intervention, spontaneous abortion, where the fetus passes away before 28 weeks of pregnancy due to medical intervention, is medically indicated abortion. However, stillbirth was defined as the birth of a child who passed away at any time between 28 and pregnancy through to the due date of birth [17].

Neonatal mortality: Neonates die within 28 days of birth [19].

Preterm birth is if the baby is born before 37 completed weeks of gestation but after 28 weeks of gestation [19].

Low birth weight, is when the newborn weight is less than 2500 gm. within the first hour of birth [19].

### Data collection tool and procedures

Data for the HDSS survey was gathered using a structured questionnaire that was administered by an interviewer. The questionnaire covers information on house characteristics, birth, death, in-migration, out-migration, pregnancy observation, pregnancy outcomes, and vaccination status. Currently, about 22 data collectors and 5 supervisors are at work collecting data and supervising nine kebeles. The data was gathered using the Open Data Kit [ODK] app on a smartphone during in-person interviews. Every home is visited by data collectors twice a year, and women are asked about their pregnancies and the results of those pregnancies.

During the twice-yearly field visits, the data collectors bring the list of household inhabitants up to date. Before sending the information to the database system, field managers verified its accuracy. Supervisors instructed the data gatherers to return and correct any errors they discovered. Data was gathered on a tablet computer in the field, and it was briefly saved on ODK aggregate. Once the data manager gave his approval, the information was moved from interim storage to the final "Openhds" database system. All data was fully anonymized before we accessed it by the data clerk.

### Data analysis procedures

Data analysis was performed using STATA version 17. Pregnancy data were first merged with individual socio-demographic data using mother IDs. Editing, cleansing, and data management have all been finished. Missing data is managed using multiple imputation techniques. Prevalence proportions of adverse birth outcomes were computed by dividing the total number of adverse birth outcomes by the total number of births during the study year. We examined the complete data for the years 2018 to 2022 to identify the factors linked to adverse pregnancy outcomes. Log-binomial regression in multivariate analysis was used to evaluate factors linked to unfavorable pregnancy outcomes (stillbirth and abortion or miscarriage).

The generalized linear model utilized by the log-binomial regression approach to characterize the likelihood of the result [adverse pregnancy outcome] uses the logarithm of the probability as the link function [17]. Variables in binary binomial regression with p-values less than 0.2 were included in the final model. Utilizing the prevalence proportion ratio (PPR), the strength and degree of relationship were both measured. Statistical significance was determined by a p-value of 0.05 or less.

## Results

### Socio-demographic characteristics

A total of 6664 reproductive-age women (15–49 years) with 8885 pregnancies were included in the study. Of the study participants, the majority, 6,294 [70.84%] were in the age range of 20–34 years, and the median (IQR) age of women was 28.3 (10.17) years and ranged from 12.9 to 46.7 years. More than half did (56.67%) not attend formal education. The majority of these women, 5684 [63.97%], were multipara, and regarding the parity, over half [56.18%] reported 1–3. Nearly 3/4th, 72.81%, of the respondents, attended ANC and nearly 60% got the tetanus toxoid (TT) vaccine. Only 5.58% of the study subjects reported a history of pregnancy loss. When it comes to the sex of neonates, 49.42% were boys, and the majority of deliveries take place at home [**Table 1**].

**Table 1:** Socio-demographic and maternal characteristics of the study participating reproductive age women included in HDSS at Arba Minch Zuria and Gacho Baba districts, southern Ethiopia, 2018–2022, n = 8885.

Variables	Category	Frequency	Percentage [%]
Age in year	<20	842	9.48
	20–34	6,294	70.84
	>34	1,749	19.68
Educational status	Illiterate	5,035	56.67
	Literate	3,850	43.33
Gravidity	Primigravida [1]	1620	18.23
	Multipara [2–4]	5684	63.97
	Grand multipara[≥5]	1,581	17.79
Parity	Nulliparous	432	4.86
	1–3	4,992	56.18
	≥4	3,461	38.95
ANC attendances	Yes	6,469	72.81
	No	2,416	27.19
History of pregnancy loss	Yes	496	5.58
	No	8,389	94.42
Sex of neonates	Male	4,391	49.42
	Female	4,494	50.58
Receive tetanus toxoid (TT) vaccine	Yes	5,302	59.67
	No	3,583	40.33
Place of delivery	Home	4,861	54.71
	Institution	4,024	45.29

### Pregnancy loss rate

**Fig 1:**
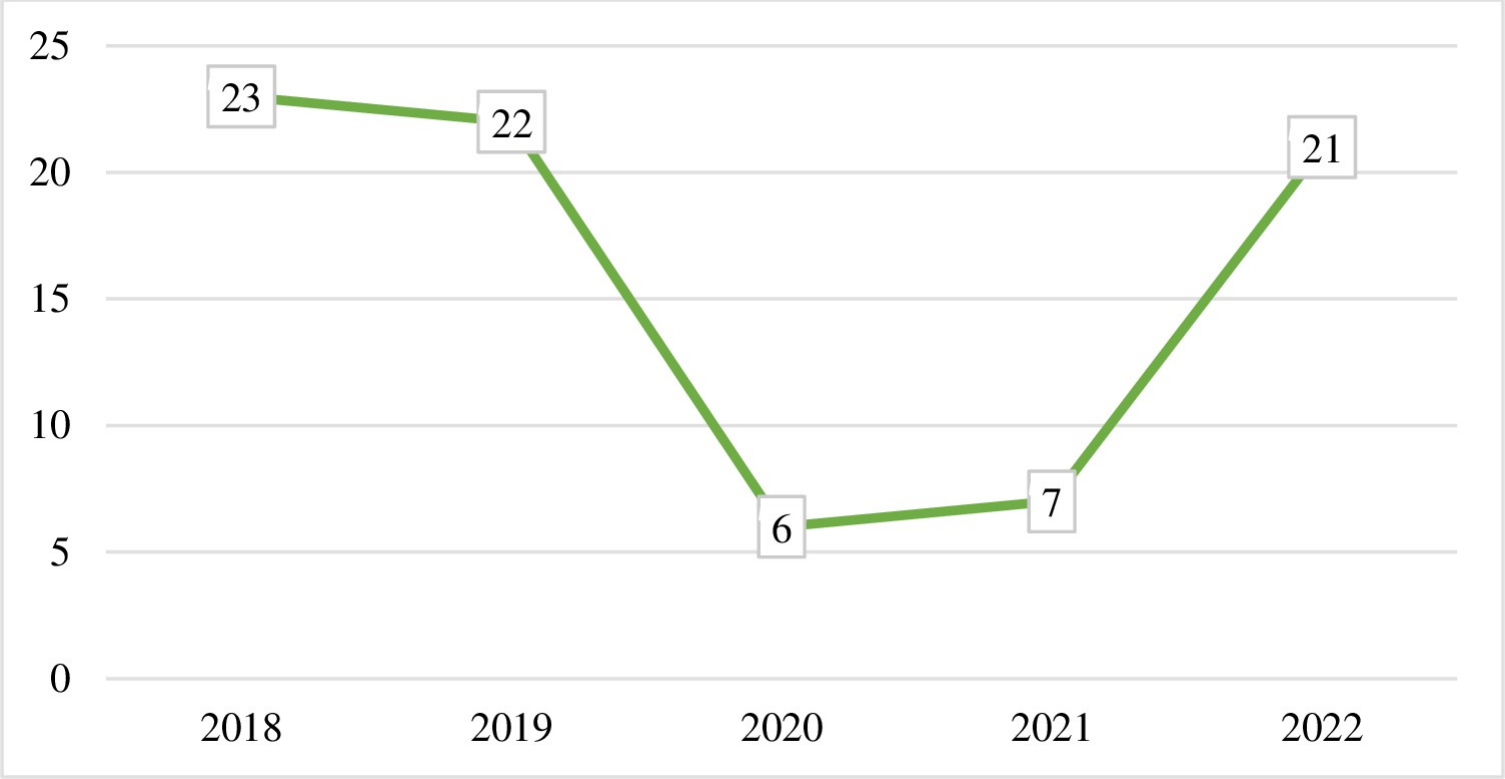
Trends of pregnancy loss among reproductive age women from Arba Minch Zuria woreda, southern Ethiopia, 2018–2022, n = 8885.

**Fig 2:**
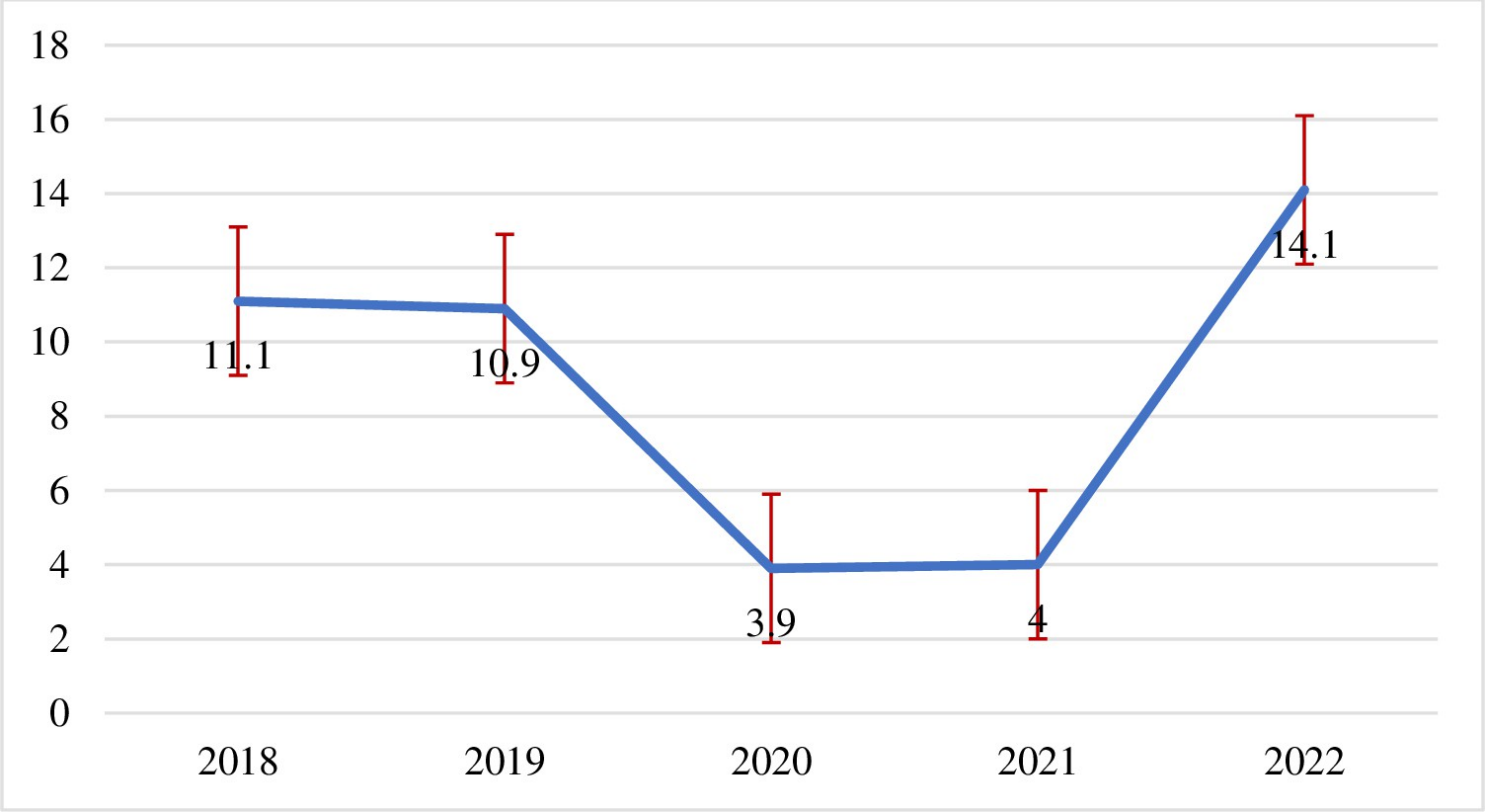
Trends and rate pregnancy lost per 1000 Arba Minch Zuria and Gacho Baba districts, southern Ethiopia, 2018–2022, n = 8885.

From 8885 pregnancies from 2018 to 2022, 51 [0.57%] resulted in stillbirth, 24 [0.27%] miscarriage, and 4 (0.04%) abortion abortions. A larger number of births [2,068/8885] and stillbirths [18/51] were recorded in 2018. Total stillbirth and abortion rates were 5.74 per 1000 births [95%CI: 4.36, 7.54] and 3.15 per 1000 births [95%CI: 1.97, 4.02] respectively. The overall adverse pregnancy outcome was 8.89 per 1000 live births [95%CI: 6.93, 10.84]. And the trends of adverse pregnancy outcomes showed 11.1/1000 in 2018 and 14.1/1000 in 2022 **(Figs 1, 2)**.

### Determinants of adverse pregnancy outcomes

In our study, there are three predictors associated with adverse pregnancy outcome outcomes; maternal age, antenatal care (ANC), and history of previous pregnancy loss. As shown in Table 2, the proportion of adverse pregnancy outcomes is around 3 times higher in those pregnant mothers older than 34 years, compared to 20 to 34 year old pregnant mothers [aPPR 2.93, 95% CI 1.67, 5.17]. Pregnant women who attended ANC were 48% less likely to develop adverse pregnancy outcomes compared to pregnant mothers who did not attend ANC follow-up [aPPR 0.52, 95% CI 0.33, 0.82]. The proportion of adverse pregnancy outcomes is around 2.7 times higher in those mothers who have a history of pregnancy loss compared to those who have no history of pregnancy loss [aPPR 2.68, 95% CI 1.36, 5.29] **(Table 2)**.

**Table 2:** Factors associated with adverse pregnancy outcomes of the study participating reproductive age women included in HDSS at Arba Minch zuria and Gacho Baba districts, southern Ethiopia, 2018–2022, n = 8885.

Variables	Category	Adverse pregnancy outcomes		PPR [95%CI]	aPPR [95%CI]
		Yes	No		
Age in year	20–30	35[0.56]	6259[99.44]	1	1
	<20	11[1.31]	831[98.69]	2.24[2.21,2.26]	2.39[2.23,2.56] **
	>34	33[1.89]	1716[98.11]	3.12[3.09,3.14]	2.41[2.23,2.61] **
Gravidity	Primigravida [1]	16[0.99]	1,604 [99.01]	1	1
	Multipara [2–4]	36[0.63]	5,648 [99.37]	0.83[0 .760,0.91]	1.01[0.90,1.14]
	Grand multipara[≥5]	27[1.71]	1554[98.29]	2.09[1.89,2.30]	1.66[1.40,1.98] **
ANC	Yes	47[0.73]	6,422[99.27]	0.68[0.675,0.685]	0.67[0.66, 0.68]
	No	32[1.32]	2,384[98.68]	1	1
History pregnancy loss	Yes	10[2.02]	486[97.98]	2.12[1.85, 2.43]	2.11[1.84, 2.43]
	No	69[0.82]	8320[99.18]	1	1

## Discussion

The overall unfavorable pregnancy outcome was nearly 9 per 1000 pregnancies, and the prevalence shows variation over time, the 2022 result shows increments compared to the rest of the years. In addition, maternal age, antenatal care, and history of pregnancy loss were factors associated with it.

In our study, a pregnant mother whose age is beyond 34 years becomes a risk factor for adverse outcomes. This research is comparable with the result of meta-analysis and systematic review showing that mothers whose age is greater than 35 years were associated with a 75% increment in risk of having stillbirth [20], and another paper also shows 18% higher risk [21]. Additionally, pregnancy conceived at a late age has been shown to increase the risk of miscarriage [22] and abortion [23]. The reason for this increased risk may be that older women may have more pre-medical conditions like preeclampsia, hypertension, and gestational diabetes [21, 22]. Additionally, women of older age are more prone to developing fetal growth restriction [24] which can most probably lead to stillbirth and chromosomal abnormalities in a fetus [22]. Contrary to other studies [25], our research found no evidence of a substantial association between young pregnancy and poor pregnancy outcome outcomes. Differences in study area and sample size may be possible justifications for this.

Another result in our study indicated that attending antenatal care [ANC] becomes a preventive factor for unwanted pregnancy outcomes. Our finding is buoyed by the research carried out in Kenya and Ethiopia. Research conducted in Kenya indicated women who did not attend ANC follow-up had a four times increased chance of having a stillbirth. Another study done in Ethiopia indicated that quality ANC lowers the odds of stillbirth by 81% by adjusting other confounders. Antenatal care not only prevents stillbirth but also can prevent miscarriage. One study conducted in Ghana revealed that ANC visits can lower the risk of miscarriage by 43% [26]. The reason for such a decrease in unwanted pregnancy outcomes may be attributed to ANC services provided for pregnant women, including direct preventive activities like screening and management of pre-eclampsia, gestational diabetics, and monitoring of fetal growth and well-being, and these services may prevent unwanted consequences, and it promotes skilled healthcare care provision and healthcare facility deliveries [27, 28].

This study found that a history of pregnancy loss is a significant predictor for unwanted pregnancy outcomes. This is comparable with results from studies conducted in Ethiopia [29], Iran [30], the United States [31], Germany [32], and China [33]. The association between a history of pregnancy loss and unwanted pregnancy outcomes may be brought on by underlying health conditions, genetic predispositions, environmental factors, infections, and low quality of life. However, all studies did not have the same conclusions as the above. For example, studies conducted in Ethiopia [34, 35], Sudan [36], and China [37] have not found a significant association between a history of pregnancy loss and adverse pregnancy outcomes. These discrepancies in the study design, sample size, and measurement error could be the possible reasons for such inconsistency.

Our paper utilized a large sample and five years of data, which can indicate the negative pregnancy outcome condition in the area, which can be one of the strengths of this study. One of the main limitations of this study was that variables such as comorbidities, income, and behavioral factors, which can be associated with adverse pregnancy outcomes, were not included in the data.

The analysis of health and demographic surveillance data from Arba Minch Zuria and Gacho Baba Woredas revealed a concerning trend in adverse pregnancy outcomes over four years from 2018 to 2022. The rate of adverse pregnancy outcomes increased from 11.1 per 1000 live births in 2018 to 14.1 per 1000 live births in 2022, indicating an increase in the prevalence of complications during pregnancy and childbirth in these regions.

This increasing trend in adverse pregnancy outcomes raises several important considerations for health care providers, policymakers, and public health practitioners. Firstly, it underscores the need for targeted interventions to address the underlying factors contributing to adverse pregnancy outcomes in Arba Minch Zuria and Gacho Baba Woredas. These interventions should focus on improving access to quality antenatal care, promoting maternal nutrition and health education, ensuring skilled attendance at birth, and addressing social determinants of health that impact pregnancy outcomes. Inadequate data to describe further by disaggregate stillbirth and abortion was a limitation.

## Conclusion and recommendation

In conclusion, the rate of adverse pregnancy outcomes decreased from 2018 to 2020, showed a slight increment in 2021, and was even higher in 2022. This adverse pregnancy outcome is associated with higher maternal age, ANC follow-up, and previous pregnancy loss.

The findings of this research emphasize the importance of considering the risks linked to pregnancy at an older age and the importance of regular prenatal care and early intervention for both the mother and the newborn.

Based on our findings, a late pregnancy can result in adverse pregnancy outcomes. Consequently, these mothers in this age group should receive professional support regarding their reproductive choices and decisions. Moreover, pregnant women should have regular ANC follow-ups for optimal maternal and fetal outcomes.

These findings could be of some help to policies and programs fighting against adverse birth outcomes and targeting maternal and newborn health in Southern Ethiopia.

## Supporting information

S1 Data (DTA)

## References

[1] World Health Organization, Managing complications in pregnancy and childbirth: a guide for midwives and doctors– 2nd ed. Geneva: 2017. License license license: CC BY-NC-SA 3.0 IGO.

[2] Kramer MS. The epidemiology of adverse pregnancy outcomes: an overview. J Nutr. 2003; 133(5 Suppl 2):1592S–6S. doi: 10.1093/jn/133.5.1592S 12730473

[3] UNICEF, WORLD< BANK, UN. A Neglected Tragedy The global burden of stillbirths. 2020.

[4] Agampodi S, Hettiarachchi A, Agampodi T. Making miscarriage matter. Lancet. 2021; 398(10302):745. doi: 10.1016/S0140-6736(21)01426-4 34454668

[5] Bearak J, Popinchalk A, Ganatra B, Moller AB, Tuncalp O, Beavin C, et al. Unintended pregnancy and abortion by income, region, and the legal status of abortion: estimates from a comprehensive model for 1990–2019. Lancet Glob Health. 2020; 8(9):e1152–e61. doi: 10.1016/S2214-109X(20)30315-6 32710833

[6] Tamirat KS, Sisay MM, Tesema GA, Tessema ZT. Determinants of adverse birth outcome in Sub-Saharan Africa: analysis of recent demographic and health surveys. BMC Public Health. 2021; 21(1):1092. doi: 10.1186/s12889-021-11113-z 34098914 PMC8186187

[7] Dallak FH, Gosadi IM, Haidar WN, Durayb AA, Alomaish AR, Alshamakhi AH, et al. Prevalence of adverse birth outcomes and associated factors in Jazan, Saudi Arabia: A cross-sectional study. Medicine (Baltimore). 2022; 101(41):e31119. doi: 10.1097/MD.0000000000031119 36254006 PMC9575805

[8] Patel KK, Saroj RK, Kumar M. Prevalence and determinants of adverse pregnancy outcomes among women in India: a secondary data analysis. Indian Journal of Community Medicine. 2021 Jul 1;46(3):434–7. 34759482 10.4103/ijcm.IJCM_569_20 PMC8575229

[9] Abadiga M, Mosisa G, Tsegaye R, Oluma A, Abdisa E, Bekele T. Determinants of adverse birth outcomes among women delivered in public hospitals of Ethiopia, 2020. Arch Public Health. 2022; 80(1):12. doi: 10.1186/s13690-021-00776-0 34983656 PMC8728986

[10] Kassahun EA, Mitku HD, Getu MA. Adverse birth outcomes and its associated factors among women who delivered in North Wollo zone, northeast Ethiopia: a facility based cross-sectional study. BMC Res Notes. 2019; 12(1):357. doi: 10.1186/s13104-019-4387-9 31234898 PMC6591928

[11] Tadese M, Dagne K, Wubetu AD, Abeway S, Bekele A, Misganaw Kebede W, et al. Assessment of the adverse pregnancy outcomes and its associated factors among deliveries at Debre Berhan Comprehensive Specialized Hospital, Northeast Ethiopia. PLoS One. 2022; 17(7):e0271287. doi: 10.1371/journal.pone.0271287 35802663 PMC9269379

[12] National Vital Statsitica Report, Fetal Mortality: United States, 2020. 2020. Contract No.: 4.

[13] Kim MK, Lee SM, Bae SH, Kim HJ, Lim NG, Yoon SJ, et al. Socioeconomic status can affect pregnancy outcomes and complications, even with a universal healthcare system. Int J Equity Health. 2018; 17(1):2. doi: 10.1186/s12939-017-0715-7 29304810 PMC5756361

[14] Bai G, Korfage IJ, Mautner E, Raat H. Associations between Maternal Health-Related Quality of Life during Pregnancy and Birth Outcomes: The Generation R Study. Int J Environ Res Public Health. 2019; 16(21). doi: 10.3390/ijerph16214243 31683775 PMC6862207

[15] Yeoh PL, Hornetz K, Shauki NIA, Dahlui M. Evaluating the quality of antenatal care and pregnancy outcomes using content and utilization assessment. Int J Qual Health Care. 2018; 30(6):466–71. doi: 10.1093/intqhc/mzy041 29590356

[16] Tsegaye Berhan and Kassa Andargachew, Prevalence of adverse birth outcome and associated factors among women who delivered in Hawassa town governmental health institutions, south Ethiopia, in 2017. 10.1186/s12978-018-0631-3 PMC6260556 30477512

[17] MacDorman MF, Gregory EC. Fetal and perinatal mortality: United States, 2013. Natl Vital Stat. 2015; 64[8]:1–24. 26222771

[18] Skove T, Deddens J, Petersen MR, Endahl L. Prevalence proportion ratios estimation and hypothesis testing. Int J epidemiology. 1998; 27[1]:91–5. 10.1093/ije/27.1.91 9563700

[19] Tadese Mesfin, et al, Assessment of the adverse pregnancy outcomes and its associated factors among deliveries at Debre Berhan Comprehensive Specialized Hospital, Northeast Ethiopia,2022. 10.1371/journal.pone.0271287 PMC9269379 35802663

[20] Lean SC, Derricott H, Jones RL, Heazell AEP. Advanced maternal age and adverse pregnancy outcomes: A systematic review and meta-analysis. PLoS One. 2017 Oct 17;12(10):e0186287. doi: 10.1371/journal.pone.0186287 ; PMCID: PMC5645107. 29040334 PMC5645107

[21] Shekari M., Shirzadfardjahromi M., Ranjbar A., Mehrnoush V., Darsareh F., & Roozbeh N. (2022). Advanced maternal age and adverse obstetrical and neonatal outcomes of singleton pregnancies. *Gynecology and Obstetrics Clinical Medicine*, 2(4), 175–180. 10.1016/j.gocm.2022.10.004.

[22] Londero A.P., Rossetti E., Pittini C. et al. Maternal age and the risk of adverse pregnancy outcomes: a retrospective cohort study. BMC Pregnancy Childbirth 19, 261 (2019). doi: 10.1186/s12884-019-2400-x 31337350 PMC6651936

[23] Zhang M, Yang BY, Sun Y, Qian Z, Xaverius PK, Aaron HE, et al. Non-linear Relationship of Maternal Age With Risk of Spontaneous Abortion: A Case-Control Study in the China Birth Cohort. Front Public Health. 2022 Jul 14;10:933654. doi: 10.3389/fpubh.2022.933654 ; PMCID: PMC9330030. 35910867 PMC9330030

[24] Lean S. C., Heazell A. E., Dilworth M. R., Mills T. A., & Jones R. L. (2017). Placental Dysfunction Underlies Increased Risk of Fetal Growth Restriction and Stillbirth in Advanced Maternal Age Women. Scientific Reports, 7(1), 1–16. 10.1038/s41598-017-09814-w. 28852057 PMC5574918

[25] Wu Q. J., Li Y., Wang L., & Wu L. (2020). Teenage Pregnancy and Adverse Birth Outcomes: A Systematic Review and Meta-Analysis. Journal of Pediatric and Adolescent Gynecology, 33(5), 441–450.32981590

[26] Chireh B, Essien SK, D’Arcy C. trimester first-trimester antenatal care visit reduces the risk of miscarriage among women of reproductive age in Ghana, Afr J Reprod Health 2021; 25[1]: 129–137) doi: 10.29063/ajrh2021/v25i1.15 34077119

[27] Zhang et al. The adverse maternal and perinatal outcomes of adolescent pregnancy: a cross-sectional study in Hebei, China BMC Pregnancy and Childbirth (2020) 20:339. 32487101 10.1186/s12884-020-03022-7 PMC7268722

[28] World Health Organization (WHO). WHO Recommendations on Antenatal Care for a Positive Pregnancy Experience: Summary. Geneva, Switzerland: WHO; 2018. License: CC BY-NC-SA 3.0 IGO. 28079998

[29] Yemane A., et al., Gestational hypertension and progression towards preeclampsia in Northern Ethiopia: prospective cohort study. BMC Pregnancy and Childbirth, 2021. 21: p. 1–8. 33784971 10.1186/s12884-021-03712-w PMC8008690

[30] Sepidarkish M., et al., Association between previous spontaneous abortion and pre‐eclampsia during a subsequent pregnancy. International Journal of Gynecology & Obstetrics, 2017. 136[1]: p. 83–86. doi: 10.1002/ijgo.12008 28099708

[31] Lazarides, C.A.-O., et al., The association between history of prenatal loss and maternal psychological state in a subsequent pregnancy: an ecological momentary assessment [EMA] study. [1469–8978 [Electronic]]. 10.1017/S0033291721002221 PMC9975992 34127159

[32] Ahrens K.A., Rossen L.M., and Branum A.M., Pregnancy loss history at first parity and selected adverse pregnancy outcomes. [1873–2585 [Electronic]]. 10.1016/j.annepidem.2016.04.011 PMC6626662 27262817

[33] Yang J., et al., Adverse Pregnancy Outcomes of Patients with History of First-Trimester Recurrent Spontaneous Abortion. BioMed Research International, 2017. 2017: p. 4359424. doi: 10.1155/2017/4359424 28798930 PMC5536133

[34] Tsegaye B. and Kassa A., Prevalence of adverse birth outcome and associated factors among women who delivered in Hawassa town governmental health institutions, south Ethiopia, in 2017. Reproductive Health, 2018. 15[1]: p. 193. doi: 10.1186/s12978-018-0631-3 30477512 PMC6260556

[35] Kebede A.S., Muche A.A., and Alene A.G., Factors associated with adverse pregnancy outcome in Debre Tabor town, Northwest Ethiopia: a case-control study. BMC Research Notes, 2018. 11[1]: p. 820. doi: 10.1186/s13104-018-3932-2 30454020 PMC6245821

[36] Mohamedain A., et al., Association between previous spontaneous abortion and preeclampsia: a case-control study. BMC Pregnancy and Childbirth, 2022. 22[1]: p. 715. doi: 10.1186/s12884-022-05053-8 36123591 PMC9484178

[37] Lao T.T., et al., Prior abortion history and pregnancy hypertensive disorders in primiparous gravidae. Pregnancy Hypertension, 2018. 14: p. 168–173. doi: 10.1016/j.preghy.2018.10.001 30527107

